# Supplementary material for: Robust estimation of bacterial cell count from optical density
Source: Commun Biol. 2020 Sep 17;3:512. doi: 10.1038/s42003-020-01127-5 (PMC7499192; doi:10.1038/s42003-020-01127-5)
Supplement: Supplementary file 6 — Description of Additional Supplementary Files [file 42003_2020_1127_MOESM6_ESM.pdf]

Description of Additional Supplementary Files

File Name: **Supplementary Data 1**
Description: File containing DNA constructs for the 2018 iGEM Interlab Study.

File Name: **Supplementary Data 2**
Description: JSON files containing of all input data sets, plus all results of analysis per Methods above. Team names are omitted in order to anonymize data sets. For flow cytometry data, only the per-sample statistical summary of each sample is included.

File Name: **Supplementary Data 3**
Description: Spreadsheet indicating the number of team datasets with valid data points included for each construct and calibration / measurement condition in Figures 4 and 5.
